# Meningococcal Factor H Binding Protein fHbpd184 Polymorphism Influences Clinical Course of Meningococcal Meningitis

**DOI:** 10.1371/journal.pone.0047973

**Published:** 2012-10-23

**Authors:** Jurgen R. Piet, Matthijs C. Brouwer, Rachel Exley, Stijn van der Veen, Diederik van de Beek, Arie van der Ende

**Affiliations:** 1 Department of Neurology, Center of Infection and Immunity Amsterdam (CINIMA), Academic Medical Center, University of Amsterdam, Amsterdam, The Netherlands; 2 Department of Medical Microbiology, Center of Infection and Immunity Amsterdam (CINIMA), Academic Medical Center, University of Amsterdam, Amsterdam, The Netherlands; 3 Sir William Dunn School of Pathology, University of Oxford, Oxford, United Kingdom; 4 Netherlands Reference Laboratory for Bacterial Meningitis, Amsterdam, The Netherlands; Public Health Ontario, Canada

## Abstract

Factor H Binding protein (fHbp) is an important meningococcal virulence factor, enabling the meningococcus to evade the complement system, and a main target for vaccination. Recently, the structure of fHBP complexed with factor H (fH) was published. Two fHbp glutamic acids, E_283_ and E_304_, form salt bridges with fH, influencing interaction between fHbp and fH. Fifteen amino acids were identified forming hydrogen bonds with fH. We sequenced *fHbp* of 254 meningococcal isolates from adults with meningococcal meningitis included in a prospective clinical cohort to study the effect of fHbp variants on meningococcal disease severity and outcome. All fHbp of subfamily A had E304 substituted with T304. Of the 15 amino acids in fHbp making hydrogen bonds to fH, 3 were conserved, 11 show a similar distribution between the two fHbp subfamilies as the polymorphism at position 304. The proportion of patients infected with meningococci with fHbp of subfamily A with unfavorable outcome was 2.5-fold lower than that of patients infected with meningococci with fHbp of subfamily B (2 of 40 (5%) vs. 27 of 213 (13%) (*P* = 0.28). The charge of 2 of 15 amino acids (at position 184 and 306) forming hydrogen bonds was either basic or acidic. The affinity of fHbp_K184_ and of fHbp_D184_ for recombinant purified human fH was assessed by Surface Plasmon Resonance and showed average K_D_ of 2.60×10^−8^ and 1.74×10^−8^, respectively (ns). Patients infected with meningococci with fHbp_D184_ were more likely to develop septic shock during admission (11 of 42 [26%] *vs.* 19 of 211 [9%]; *P = *0.002) resulting in more frequent unfavorable outcome (9 of 42 [21%] *vs.* 20 of 211 [10%]; *P* = 0.026). In conclusion, we dentified fHBP_D184_ to be associated with septic shock in patients with meningococcal meningitis.

## Introduction

*Neisseria meningitidis* (the meningococcus) is the leading cause of meningitis and septicemia in young adults, and is associated with substantial mortality and morbidity [1]–[3]. The meningococcus is a commensal of the human upper respiratory tract with asymptomatic colonization occuring in up to 30% of healthy individuals [4]–[7]. Invasive meningococcal disease has been associated with environmental factors like smoking, living in the same household as a patient and disease in proxies [8]. Other important risk factors for developing disease can be found in bacterial and host genetic factors [9], [10]. The influence of genetic bacterial factors is illustrated by a higher abundance of hypervirulent clones among isolates from patients as compared to asymptomatic carriers [11]. The distribution of meningococcal housekeeping genes has also been described to influence disease course in patients with meningococcal meningitis. Meningococci of clonal complex 11 (cc11) were associated with sepsis and unfavourable outcome [2]. Furthermore, meningococci with penta-acylated instead of hexa-acylated lipid A (due to a mutation in the *lpxL1* gene) were found to activate TLR4 less efficiently, leading to reduced inflammation and coagulopathy [12]. On the host side, case control studies found single nucleotide polymorphisms (SNPs) influencing susceptibility and outcome of meningococcal disease [13], [14]. A genome wide association study has recently identified an SNP in human complement downregulator factor H (fH) to be associated with susceptibility [9].

Activation of the complement cascade is a critical host defense against meningococcal disease [15]. Many pathogenic organisms have found ways to elude complement attack [16]. Meningococcal fH binding protein (fHbp) binds fH, thus inhibiting complement activation and hampering complement-mediated lysis in human plasma [17], [18]. This 27 kDa lipoprotein, formerly named genome-derived neisserial antigen 1870 (GNA1870) [19] or lipoprotein 2086 [20], is present on the surface of all meningococcal strains [20]. It acts as a receptor for fH, although fHbp is not the only factor determining the amount of fH bound [21], [22]. High levels of fHbp has been found in hypervirulent meningococcal strains [19]. Experiments with *fHbp* knock-out meningococcal strains showed a normal growth in broth, but no survival in blood [23]. Some strains have incomplete (truncated) forms of *fHbp*, but the functional relevance is unknown [24]. Inhibiting the binding of fH to the neisserial surface by vaccine derived fHbp antibodies may enhance bactericidal activity of the complement system, resulting in two distinct mechanisms of antibody-mediated vaccine efficacy [17].

Epidemiological surveys showed a high *fHbp* sequence diversity leading to great variations in fHbp structure. Currently, over 550 fHbp protein types have been identified (http://pubmlst.org/neisseria/fHbp/). These protein types can be subdivided in 3 major fHbp subgroups: variant 1 (subfamily B), and variants 2 and 3 (collectively called subfamily A) [19], [20]. The overall fHbp architecture appears to be modular, consisting of combinations of five modular variable segments, each flanked by blocks of two to five conserved amino acids [25]. Each of the modular segments is derived from one of two lineages and based on the modular classification fHbp can be classified in six modular groups (I–VI). Recently, the structure of fHbp of subfamily B in complex with fH was published [18]. Two fHbp amino acids, E_283_ and E_304_, were found to be important for the interaction with fH, via salt bridges. A further fifteen amino acids were identified hydrogen bonding to fH.

Results of studies of *fHbp* variance among patients’ isolates can have important consequences for further vaccine development as inclusion of more fHbp proteins could improve vaccine efficacy. Furthermore, it is unclear if the variation in *fHbp* and its encoded protein influences disease characteristics and outcome. Therefore we investigated the distribution of fHbp protein variants among the meningococcal isolates of 254 patients with meningitis from a nation-wide prospective cohort study and correlate protein types with clinical characteristics.

## Results

### Cohort of Patients with Meningococcal Meningitis

From October 1998 to April 2002, 258 episodes of community-acquired meningococcal meningitis in 258 patients were included. Detailed clinical and microbiological characteristics have been described previously [2], [26]. Patient characteristics, clinical course and the causative bacterial strain isolated from the cerebrospinal fluid (CSF) were available for 254 (98.4%) of the 258 meningococcal meningitis episodes. The case fatality rate was 7%, and 12% patients had an unfavorable outcome, defined as a score from one to four on the Glasgow outcome scale [27].

### Multi Locus Sequence Typing and fHbp Sequencing

Serogrouping results were available for all 254 meningococcal strains and have been described previously [2]. Of these, 172 (68%) were of serogroup B, 78 (31%) of serogroup C, 3 (1%) of serogroup Y, and 1 (<1%) was of serogroup W135. MLST data of 254 isolates showed 91 unique sequence types (ST). The most prevalent clonal complexes (cc) were cc41/44 (41%), cc11 (24%), and cc32 (16%). All cc11 strains were serogroup C [2]. One isolate contained an 143 bp duplication in *fHbp* resulting in a premature stop codon and was excluded as non-functional. Full length *fHbp* sequences could be obtained from the 253 meningococcal strains. DNA and protein allele numbers were assigned by the *Neisseria* Factor H binding protein sequence typing website (http://pubmlst.org/neisseria/fHbp). All meningococcal serogroup data, sequence types, clonal complexes, and fHbp DNA/protein allele are shown in Table S1.

### fHbp Protein Type Prevalence

Among 253 isolates, 42 unique *fHbp* sequences were found, of which 40 encoded unique protein sequences of mature fHbp. Of 253 fHbp protein sequences, 212 (84%) could be assigned to subfamily B (nomenclature according to Fletcher *et al* [20]) or variant 1 (nomenclature according to Masignani *et al* [19]). Of these 212 isolates 201 were of modular group I (nomenclature according to Beernink and Granhoff [25]) and 11 of modular group IV. Forty isolates (16%) belonged to subfamily A of which 32 belong to variant 2 (13%) and 8 to variant 3 (3%; Table 1). The isolates with subfamily A fHbp comprised modular groups II, III, V and VI, with 3, 12, 5 and 20 isolates, respectively. Strain 2011148 contained an intermediate fHbp with sequences from subfamily A and B. Of 161 serogroup B isolates, 15 had subfamily A fHbp, 155 had subfamily B and one had a hybrid A/b fHbp. Of 78 serogroup C isolates, 21 had subfamily A fHbp and 57 had subfamily B fHbp. The one serogroup W135 and the three serogrop Y isolates had subfamily A fHbp (Table S1). The major hypervirulent clonal complexes cc41/44, cc32 and cc11 had predominantly subfamily B fHbp or variant 1∶98 of 104 (94%), 39 of 40 (98%) and 50 of 62 (81%), respectively. Isolates of cc8 had predominantly fHbp of subfamily A: 9 of 10 (90%; all variant 2) (Table 1). The most prevalent fHbp protein types (protein type from the fHbp database at http://pubmlst.org/neisseria/fHbp/) were 14 (35%), 1 (15%) and 10 (10%). They were strongly associated with meningococcal sequence type: Of 89 isolates with fHbp protein type 14, 87 (98%) were cc 41/44, 38 of 38 (100%) of fHbp type 1 were cc32 and 25 of 26 fHbp type 10 isolates were from cc11 (Table 2).

**Table 1:** Distribution of meningococcal fHbp subfamilies and variants among clonal complexes.

	fHbp subfamily^a^			
	A		B	A/B hybrid
	variant^b^		variant	
clonal complex	2	3	1	
cc11	10	2	50	
cc167	1			
cc174	1			
cc18			2	
cc213		1		
cc22	1			
cc269	2	1	13	
cc32		1	39	
cc35	1			
cc364	1			
cc41/44	4	1	98	1
cc461		1	1	
cc60			4	
cc8	9		1	
cc92	1			
singleton	1	1	4	
all	32	8	212	1

a Nomenclature according to Fletcher *et al* ^17^.

b Nomenclature according to Masignani *et al* ^21^.

**Table 2:** fHbp protein type and clonal complexes.

		fHbp protein type^a^													
Clonal complex	No. (%)	1	4	10	13	14	15	16	19	22	25	89	129	131	Other
ST-41/44 complex/Lineage 3	104 (41)		5	1	1	87			3						7
ST-11 complex/ET-37 complex	62 (24)		1	25					1	5	3	3	11	3	10
ST-32 complex/ET-5 complex	40 (16)	38													2
ST-269 complex	16 (6)				1		11		2						2
ST-8 complex/Cluster A4	10 (4)					1		9							
ST-60 complex	4 (2)				4										
Non typable	6 (2)		1		2	1			1						1
Other	11 (4)							2							9
Total (%)	253 (100)	38 (15)	7 (3)	26 (10)	8 (3)	89 (35)	11 (4)	11 (4)	7 (3)	5 (2)	3 (1)	3 (1)	11 (4)	3 (1)	31 (13)

a fHbp protein type from the fHbp database at http://pubmlst.org/neisseria/fHbp/.

### Associations of fHbp Polymorphism with Clinical Characteristics

Of the two residues in fHbp, E283 and E304, which are involved in salt bridges to fH and found to be important for the interaction with fH [18], E283 is conserved among fHbp of all 253 isolates. All fHbp of subfamily A (variant 2 and 3) had E304 substituted with T304. Of the 15 amino acids in fHbp making hydrogen bonds to fH [18], 3 were conserved: K264 (all except one isolate), D266 and V272. Of the 12 amino acids changed, 11 show a similar distribution between the two fHbp subfamilies as the polymorphism at position 304 if change in charge or size of side groups were considered (Table S2). The hybrid fHbp of strain 2011148 contained fHbp_E304_ and 3 of 10 of the other subfamily A polymorphisms. The results by Seib and colleagues [22] who investigated the binding of fH to purified fHbp of 12 different protein types (6 of each subfamily A and B) showed a 5-fold higher thermodynamic dissociation constant K_D_ for the interaction between fH and fHbp of subfamily B than for that between fH and fHbp of subfamily A (Table S3). The average K_D_’s were 153 nM and 29 nM, respectively (*P* = 0.02, calculated from Table 3 in [22]), demonstrating that subfamily B fHbp has a lower affinity for fH. However, physiological fH concentration is in the order of 2 µM [28], which would give rise to saturated fH-fHbp binding [23]. The difference in K_D_ is mainly caused by a 14-fold higher dissociation constant in case of fHbp of subfamily B (*P* = 0.03) (Table S3). Interestingly, the association constant of the fH-fHbp binding is 2.5-fold lower in case of fHbp of subfamily A (*P* = 0.04), indicating a slightly reduced on-rate of fH to fHbp of subfamily A. Together, these differences between fHbp of subfamily A and B prompt us to evaluate the relation between clinical course and infection with meningococci with either subfamily fHbp. Surprisingly, the proportion of patients infected with meningococci with fHbp of subfamily A (fHbp of strain 2011148 was considered to be of subfamily B) with unfavorable outcome was 2.5-fold lower than that of patients infected with meningococci with fHbp of subfamily B (2 of 40 (5%) vs. 27 of 213 (13%), though not reaching statistical significance (*P* = 0.28). Including strain 2011148 in the subfamily A group did not affect these percentages.

**Table 3:** Associations of meningococci containing fHbp_E184_ with clinical characteristics^a^.

	fHbp_D184_ (n = 42)	fHbp_H184_ or fHbp_K184_ (n = 211)	P-value
Demographics			
Age, yr (Mean ±SD)	33 (21)	37 (±18)	0.016
Duration of symptoms <24 hs	21/41 (51)	101/205 (49)	0.82
Symptoms at presentation			
Rash	26/42 (62)	136/209 (65)	0.70
Focal neurological deficits^b^	11/42 (26)	42/211 (20)	0.36
Diastolic BP (n = 242)	73 (60–80)	66 (60–90)	0.66
Blood parameters			
Positive bloodculture	22/41 (54)	106/181 (59)	0.57
Thrombocyte count, 10^9^/L (n = 240)	166 (125–226)	151 (116–185)	0.102
Creatinin µmol/L (n = 246)	95 (75–123)	94 (79–171)	0.258
Cerebrospinal fluid parameters			
CSF leukocyte count (cells/mm^3^, n = 237)^e^	5419 (1676–12478)	3500 (552–12307)	0.25
CSF/blood glucose ratio (n = 246)	0.088 (0.01–0.31)	0.02 (0.009–0.29)	0.10
Outcome parameters			
Sepsis	11/42 (26)	19/211 (9)	0.002
Unfavorable outcome (GOS = 1–4)	9/42 (21)	20/211 (10)	0.026

a Data are number/number assessed (%) or median (25th–75th percentile), unless otherwise stated.

b Defined as aphasia, monoparesis, or hemiparesis) and cranial nerve palsies.

Abbreviations: GOS  =  Glasgow Outcome Scale.

Of note, in subfamily B fHbp proteins, the residue at position 184 and 306 was either basic or acidic, whereas subfamily A fHbp proteins were basic in these positions. At postion 306 of fHbp in meningococci from patients we found either a lysine (basic) or a glutamine (acidic). Changing lysine at postion 306 in fHbp to a glutamic acid in the fHbp-fH model structure reported by Schneider and colleagues [18] may only weakly enhance the interaction between fhbp and fH through the interaction with a tyrosine at 352 in fH. Of 253 isolates, 154 had an acidic instead of a basic residue at position 306, without a correlation with the course of disease.

At position 184 of fHbp in patients’ meningococcal isolates an histidine (basic), a lysine (basic) or a aspartic acid (acidic) was found. In the published fHbp-fH model structure [18] the histidine at position 184 in fHbp is close to a loop of fH with His402, Gly403, Arg404, Lys405, Phe406, Val407. Changing Lys184 in fHbp to Asp184 may improve binding between fHbp and fH due to the interaction between the acidic Asp184 in fHbp with the basic residues Arg404 and or Lys405 in fH. We assessed the interaction between fHbp_K184_ or fHbp_D184_ with recombinant purified human fH (SCR67) by Surface Plasmon Resonance. fHbp_10 (1.P10) was chosen as a representative fHbp having an acidic residue (D, aspartic acid) at position 184. The average K_D_ was 1.74×10^−8^ M and 2.60×10^−8^ M for fHbp_D184_ and fHbp_K184_, respectively, which was not significantly different.

Of 253 isolates, 42 (41 (98%) of serogroup B and 1 (2%) of serogroup C) had a fHbp_D184_ (acidic residue at position 184), while 211 had fHbp_H184_ or fHbp_K184_ (both with a basic residue at position 184). Of the 42 isolates with fHbp_D184_, 28 (67%) belonged to cc11, while the remaining 14 isolates belonged to 6 clonal complexes; 2 were singletons. There were clear correlations with disease progression. Patients infected with meningococci with fHbp_D184_ were more likely to develop septic shock during admission (11 of 42 [26%] *vs.* 19 of 211 [9%]; *P = *0.002) contributing to a higher rate of unfavorable outcome (9 of 42 [21%] *vs.* 20 of 211 [10%]; *P* = 0.026; table 3). Of 34 patients infected with cc11 meningococci with fHbp_H184_ or fHbp_K184_ (all of subfamily B), 4 of 34 (12%) developed sepsis during clinical course as compared to 10 of 28 (36%) among the cc11 isolates with fHbp_D184_ (*P* = 0.034). In a multivariate regression analysis including age, admission score on the Glasgow coma scale, and focal neurological deficits, the association fHbp_D184_ with unfavorable outcome was preserved (odds ratio = 2.89; 95% confidence interval 1.15–7.25; *P* = 0.024).

## Discussion

Over the last decade, fHbp has become of substantial interest: it is a key component in two forthcoming vaccines. Subvariant fHbp-1.1 (subfamily B), is included in the vaccine 4CMenB of Novartis, while fHbp-1.55 (subfamily B) and fHbp-3.45 (subfamily A) are included in the vaccine rLP2086 developed by Pfizer. In addition, it is recognized as an important virulence factor, essential for survival in human blood [23]. Here we show that variance in meningococcal fHbp sequences cultured from 254 meningitis patients included in a prospective nationwide cohort study was associated with disease severity and outcome. Of 254 strains evaluated in this study, only 1 did not contain functional fHbp, consistent with the attributed important role in survival of the organism in its host [17]. One hybrid variant containing sequences from both subfamily A and B was detected. An A/B hybrid isolated in 1960 in the Netherlands has been shown to bind fH [22]. The proportion of patients with unfavorable outcome was lower among patients infected with meningococci containing fHbp of subfamily A, although this did not reach significance. Patients infected with meningococci with fHbp_D184_ were more likely to develop septic shock during admission resulting in a higher rate of unfavorable outcome.

Compared to the published structure of a subfamily B fHbp [18], fHbp of subfamily A isolates in this study had a number of substitutions at positions important for the interaction with fH. E304, one of the two residues involved in salt bridges to fH, is substituted by T304 in subfamily A fHbp. Importance of these salt bridge forming amino acids in binding to fH was demonstrated with the double mutant fHbp_E283A,E304A_, having a more than two orders of magnitude reduced affinity for fH, with almost no interaction at analyte concentrations around ten times the wild-type K_D_ [18]. However, single mutants were not investigated. Of 15 amino acids making hydrogen bonds to fH, 11 are substituted with an amino acid with a different charge or a different size (mostly smaller) of the side chain in subfamily A fHbp. This also suggests that fHbp of subfamily A might have a lower affinity for fH than fHbp of subfamily B. Hence, meningococci with subfamily A fHbp will be less protected in blood of the host, resulting in less severe disease. This seems to be contradicted by the results by Seib and colleagues [22] who investigated the binding of fH to purified fHbp of 12 different protein types (6 of each subfamily A and B). They state that the interaction between fH and fHbp of subfamily B on average had a 5-fold higher dissociation constant (K_D_) than that of fH and fHbp of subfamily A, mainly due to a 13-fold higher off-rate (k_d_). It is unclear if this is compatible with the published binding structure. Moreover, under physiological conditions, *i.e.*, about 2 µM fH [28], binding of fH to fHbp is saturated and without multiplying meningococci virtually all fHbps would be occupied by fH in blood [23]. However, when meningococci divide non ligand bound fHbp will arise and the on-rate (k_a_) determines the speed with which fHbp will be occupied. Interestingly, the k_a_ of fH to fHbp binding seems to be reduced in case of subfamily A fHbp. This might reduce survival of meningococci with subfamily A fHbp in the blood. Meningococci with subfamily B fHbp will be more protected by fH binding. However, we did not observe a significant difference between the rate of unfavorable outcome in patients infected with meningococci with fHbp of subfamily A and that of patients infected with meningococci with fHbp of subfamily B. In addition, the level of survival in human sera of recombinant MC58 strains expressing the diverse subvariants did not correlate with levels of fH binding [22]. Similar results were obtained by Dunphy and colleagues showing that expression by strain H44/76 of two natural fHbp sequence variants with lower fH affinity had minimal or no effect on non-immune clearance in blood or plasma [23]. Most likely, the outcome of the interaction between meningococcal fHbp and factor H on the survival of meningococci in blood depends on a multiple factors, like expression levels of fHbp, physiological conditions of the host that may affect factor H concentrations) than simply the affinity of fHbp with factor H.

In our study, fHbp of subfamily B was found to be dominant among all major hypervirulent clonal complexes, indicating that disease is caused mainly by meningococci with fHbp of subfamily B. Patients infected with meningococci with fHbp_D184_ (acidic residue substituting a basic residue at position 184) were more likely to develop septic shock during admission resulting in a higher rate of unfavorable outcome. Substitution of H184 to D184 in the fHbp-fH model structure [18] suggest a stronger interaction between fHbp and fH. Results of binding experiments however did not show significant differences in binding affinity between fH and the different fHbp184 variants. Possibly, the binding assay might not be sensitive enough to detect differences that are biologically relevant. Alternatively, fHbp might have other still unknown functions which are modified by this substitution. A stronger interaction between fHbp_D184_ and fH would lead to better protection of meningococci with fHbp_D184_ in the blood stream, which is consistent with more severe disease in patients infected with these meningococci. Of these patients, 67% were infected with meningococci belonging to cc11, but the predictive effect of fHbp_D184_ on outcome was preserved. Among patients infected with cc11 isolates, patients infected with meningococci with fHbp_D184_ were more likely to develop sepsis than those infected with meningococci with fHbp_H184_ or fHbp_K184_. Previously, we reported cc11 meningococcal isolates to be associated with sepsis and unfavorable outcome [2]. The present results, indicate that part of the virulence of cc11 isolates might be attributed to fHbp_D184_.

During our inclusion period, routine serogroup C vaccination had not yet started in the Netherlands. Vaccinating all children and adolescents from 14 months to 19 years -starting in June 2002- has dramatically reduced serogroup C disease in the Netherlands from 31% in our cohort to an incidence of 6.5% in 2009 (http://www.amc.nl/upload/teksten/medical microbiology/nrlbm jv/jv2009site.pdf) [29]. Therefore fHbp protein type distribution will most likely have changed, warranting epidemiological surveys of a more recent period.

In conclusion, we found fHbp_D184_ to be associated with severe disease and unfavorable outcome. This study provides insights regarding the clinical relevance of this potent vaccine target that is included in two forthcoming serogroup B vaccines. Because of the natural occurring strain variation of *fHbp*, further epidemiological surveys of sequence variation of this vaccine component remains warranted.

## Materials and Methods

### Ethics Statement

This observational study with anonymous patient data was carried out in accordance with the Dutch privacy legislation. Written informed consent to use data made anonymous was obtained from the patient (if possible) or from the patient’s legal representative. The Dutch Meningitis Cohort Study was approved by the ethics committee of the Academic Medical Center in Amsterdam.

### Meningitis Cases

The Dutch Meningitis Cohort Study from 1998–2002 was a prospective nationwide observational cohort study of adults with community-acquired bacterial meningitis in the Netherlands [26]. Inclusion and exclusion criteria have been described extensively elsewhere [2], [26]. In short, patients older than 16 years, who had bacterial meningitis confirmed by culture of cerebrospinal fluid (CSF), were included from October 1998 to April 2002 after identification by the Netherlands Reference Laboratory for Bacterial Meningitis. This laboratory received CSF isolates from 85% of all patients with bacterial meningitis in the Netherlands. The treating physician was contacted, and informed consent was obtained from all participating patients or their legally authorized representatives.

Case-record forms were used to collect data on patients’ history, symptoms and signs on admission, laboratory findings at admission, clinical course, outcome and neurologic findings at discharge, and treatment. Outcome was graded according to the Glasgow Outcome Scale. A score of one on this scale indicates death; a score of two a vegetative state (the patient is unable to interact with the environment); a score of three severe disability (the patient is unable to live independently but can follow commands); a score of four moderate disability (the patient is capable of living independently but unable to return to work or school); and a score of five mild or no disability (the patient is able to return to work or school). A favorable outcome was defined as a score of five, and an unfavorable outcome as a score below. The Glasgow Outcome Scale is a well-validated instrument with good inter-observer agreement [30]. At discharge, all surviving patients underwent a neurologic examination performed by a neurologist which included the assessment of the Glasgow Outcome Scale.

*Bacterial strains* Upon reception of bacterial isolates in the Netherlands Reference Laboratory for Bacterial Meningitis, a monoculture of the causative isolate was frozen and stored at –80°C. All isolates were passaged less than 5 times. Serogrouping and genotyping by Multi Locus Sequence Typing (MLST) have previously been described [2].

### PCR and Sequencing

PCR templates were prepared by boiling ∼100 *N. meningitidis* colonies from chocolate agar plates in 200 µl of distilled H_2_O for 5 min, and then centrifuged. 1 µl of the supernatant was used in the PCR reaction (10 µl total volume). The primers used for PCR and sequencing are listed in Table S4. The amplification steps were 95°C for 5 min, 30 cycles of 95°C for 30 s, 58°C for 30 s, and 72°C for 90 s, followed by a final extension step at 72°C for 5 min. Amplicons were 1∶9 diluted and 1 µl was used in the sequencing reaction.

### Bioinformatic and Phylogenetic Analysis

Sequences were assembled and analysed using the Codoncode aligner software suite. Evolutionary history was inferred using the Neighbor-Joining method [31]. All positions containing gaps and missing data were eliminated from the dataset (Complete deletion option). There were a total of 251 positions in the final dataset. Phylogenetic analyses were conducted in MEGA4 [32].

### In vitro Mutagenesis and Cloning of fHbp

fHbp_10 (1.P10) was chosen as a representative fHbp having an acidic residue (D, aspartic acid) at position 184. The gene encoding this protein was amplified from *N. meningitdis* strain M01 240185 (serogroup B, clonal complex ST-11; kindly provided by dr. R. Borrow, Meningococcal Reference Unit, Health Protection Agency North West Laboratory, Manchester) using primers NG2145 and NG2143 (Table S4) and cloned into pET21b at NdeI-XhoI restriction sites for expression as a C-terminally 6×HIS tagged fusion protein lacking the N-terminal sequence required for lipid attachment. This plasmid was subjected to mutagenesis using the QuikChange site directed mutagenesis kit (Agilent Technologies) with primers 1P10DKF and 1P10DKR (Table S4) to produce plasmid pET21fHbp1P10_D184K_, in which codon GAT encoding acidic residue aspartic acid (D184) was replaced by codon AAA encoding basic residue, lysine (K184). All sequences were checked by sequencing.

### fHbP Purification and Interaction with fH

Following growth of B834(DE3) strains harbouring pET21fHbp1P10 or pET21fHbp1P10D184K overnight at 21°C in LB containing 1 mM IPTG fHbp, recombinant fHbp was purified by affinity chromatography using Ni-NTA Magnetic Agarose Beads (Qiagen). Interaction with recombinant purified human fH (SCR67) was assessed by Surface Plasmon Resonance using a ProteOn XPR36 (BioRad). fHbp was immobilized on a ProteOn GLM sensor chip and increasing concentrations of fH_67_ were injected over the flow channels at 40 µl/min and allowed to dissociate for 300 seconds. ProteOn manager software was used to calculate the *K*_D_.

### Statistics

The Mann-Whitney U test was used to identify differences between groups with respect to continuous variables, and dichotomous variables were compared with use of the χ^2^ test or Fischer’s Exact test. All statistical tests were two-tailed, with *P*<0.05 regarded as significant. Analyses were undertaken with PASW Statistics 18.0. We used logistic regression analysis to calculate odds ratios and 95% CI to assess the strength of the association between potential prognostic factors and the probability of an unfavorable outcome.

## Supporting Information

Table S1 **All meningococcal isolates used in the study with serogroup data, sequence types, clonal complexes, and fHbp DNA/protein allele.**
(XLS)

Table S2 **fHbp polymorphism of the 17 amino acids showing interaction with human factor H, according to fHbp subfamilies.**
(DOCX)

Table S3 **The association constant (k_a_), dissociation constant (k_d_) and the thermodynamic dissociation constants (K_D_) of the binding between fH and fHbp, according to protein family.**
(DOCX)

Table S4 **Primers.**
(DOCX)

## References

[1] Stephens DS, Greenwood B, Brandtzaeg P (2007) Epidemic meningitis, meningococcaemia, and *Neisseria meningitidis* . Lancet 369: 2196–2210. 17604802 10.1016/S0140-6736(07)61016-2

[2] Heckenberg SG, de Gans J, Brouwer MC, Weisfelt M, Piet JR, et al (2008) Clinical features, outcome, and meningococcal genotype in 258 adults with meningococcal meningitis: a prospective cohort study. Medicine (Baltimore) 87: 185–192. 18626301 10.1097/MD.0b013e318180a6b4

[3] Rosenstein NE, Perkins BA, Stephens DS, Popovic T, Hughes JM (2001) Meningococcal disease. N Engl J Med 344: 1378–1388. 11333996 10.1056/NEJM200105033441807

[4] Claus H, Maiden MC, Wilson DJ, McCarthy ND, Jolley KA, et al (2005) Genetic analysis of meningococci carried by children and young adults. J Infect Dis 191: 1263–1271. 15776372 10.1086/428590

[5] Kellerman SE, McCombs K, Ray M, Baughman W, Reeves MW, et al (2002) Genotype-specific carriage of Neisseria meningitidis in Georgia counties with hyper- and hyposporadic rates of meningococcal disease. J Infect Dis 186: 40–48. 12089660 10.1086/341067

[6] Maclennan J, Kafatos G, Neal K, Andrews N, Cameron JC, et al (2006) Social behavior and meningococcal carriage in British teenagers. Emerg Infect Dis 12: 950–957. 16707051 10.3201/eid1206.051297 PMC3373034

[7] Christensen H, May M, Bowen L, Hickman M, Trotter CL (2010) Meningococcal carriage by age: a systematic review and meta-analysis. Lancet Infect Dis 10: 853–861. 21075057 10.1016/S1473-3099(10)70251-6

[8] Gardner P (2006) Clinical practice. Prevention of meningococcal disease. N Engl J Med 355: 1466–1473. 17021322 10.1056/NEJMcp063561

[9] Davila S, Wright VJ, Khor CC, Sim KS, Binder A, et al (2010) Genome-wide association study identifies variants in the CFH region associated with host susceptibility to meningococcal disease. Nat Genet 42: 772–776. 20694013 10.1038/ng.640

[10] Brouwer MC, van de Beek D (2009) Genetics in meningococcal disease: one step beyond. Clin Infect Dis 48: 595–597. 19191645 10.1086/596708

[11] Caugant DA, Tzanakaki G, Kriz P (2007) Lessons from meningococcal carriage studies. FEMS Microbiol Rev 31: 52–63. 17233635 10.1111/j.1574-6976.2006.00052.x

[12] Fransen F, Heckenberg SG, Hamstra HJ, Feller M, Boog CJ, et al (2009) Naturally occurring lipid A mutants in *Neisseria meningitidis* from patients with invasive meningococcal disease are associated with reduced coagulopathy. PLoS Pathog 5: e1000396. 19390612 10.1371/journal.ppat.1000396 PMC2667671

[13] Brouwer MC, de Gans J, Heckenberg SG, Zwinderman AH, van der Poll T, et al (2009) Host genetic susceptibility to pneumococcal and meningococcal disease: a systematic review and meta-analysis. Lancet Infect Dis 9: 31–44. 19036641 10.1016/S1473-3099(08)70261-5

[14] Brouwer MC, Read RC, van de Beek D (2010) Host genetics and outcome in meningococcal disease: a systematic review and meta-analysis. Lancet Infect Dis 10: 262–274. 20334849 10.1016/S1473-3099(10)70045-1

[15] Lo H, Tang CM, Exley RM (2009) Mechanisms of avoidance of host immunity by *Neisseria meningitidis* and its effect on vaccine development. Lancet Infect Dis 9: 418–427. 19555901 10.1016/S1473-3099(09)70132-X

[16] Lambris JD, Ricklin D, Geisbrecht BV (2008) Complement evasion by human pathogens. Nat Rev Microbiol 6: 132–142. 18197169 10.1038/nrmicro1824 PMC2814840

[17] Madico G, Welsch JA, Lewis LA, McNaughton A, Perlman DH, et al (2006) The meningococcal vaccine candidate GNA1870 binds the complement regulatory protein factor H and enhances serum resistance. J Immunol 177: 501–510. 16785547 10.4049/jimmunol.177.1.501 PMC2248442

[18] Schneider MC, Prosser BE, Caesar JJ, Kugelberg E, Li S, Zhang Q, et al (2009) *Neisseria meningitidis* recruits factor H using protein mimicry of host carbohydrates. Nature 458: 890–893. 19225461 10.1038/nature07769 PMC2670278

[19] Masignani V, Comanducci M, Giuliani MM, Bambini S, Adu-Bobie J, et al (2003) Vaccination against *Neisseria meningitidis* using three variants of the lipoprotein GNA1870. J Exp Med 197: 789–799. 12642606 10.1084/jem.20021911 PMC2193853

[20] Fletcher LD, Bernfield L, Barniak V, Farley JE, Howell A, et al (2004) Vaccine potential of the *Neisseria meningitidis* 2086 lipoprotein. Infect Immun 72: 2088–2100. 15039331 10.1128/IAI.72.4.2088-2100.2004 PMC375149

[21] Schneider MC, Exley RM, Chan H, Feavers I, Kang YH, et al (2006) Functional significance of factor H binding to *Neisseria meningitidis* . J Immunol 176: 7566–7575. 16751403 10.4049/jimmunol.176.12.7566

[22] Seib KL, Brunelli B, Brogioni B, Palumbo E, Bambini S, et al (2011) Characterization of diverse subvariants of the meningococcal factor H (fH) binding protein for their ability to bind fH, to mediate serum resistance, and to induce bactericidal antibodies. Infect Immun 79: 970–981. 21149595 10.1128/IAI.00891-10 PMC3028832

[23] Dunphy KY, Beernink PT, Brogioni B, Granoff DM (2011) Effect of Factor H-Binding Protein Sequence Variation on Factor H Binding and Survival of *Neisseria meningitidis* in Human Blood. Infect Immun 79: 353–359. 21041484 10.1128/IAI.00849-10 PMC3019890

[24] Murphy E, Andrew L, Lee KL, Dilts DA, Nunez L, et al (2009) Sequence diversity of the factor H binding protein vaccine candidate in epidemiologically relevant strains of serogroup B *Neisseria meningitidis* . J Infect Dis 200: 379–389. 19534597 10.1086/600141

[25] Beernink PT, Granoff DM (2009) The modular architecture of meningococcal factor H-binding protein. Microbiology 155: 2873–2883. 19574307 10.1099/mic.0.029876-0 PMC2859308

[26] van de Beek D, de Gans J, Spanjaard L, Weisfelt M, Reitsma JB, et al (2004) Clinical features and prognostic factors in adults with bacterial meningitis. N Engl J Med 351: 1849–1859. 15509818 10.1056/NEJMoa040845

[27] Jennett B, Bond M (1975) Assessment of outcome after severe brain damage. Lancet 1: 480–484. 46957 10.1016/s0140-6736(75)92830-5

[28] Ingram G, Hakobyan S, Hirst CL, Harris CL, Pickersgill TP, et al (2010) Complement regulator factor H as a serum biomarker of multiple sclerosis disease state. Brain 133: 1602–1611. 20421219 10.1093/brain/awq085

[29] de Greeff SC, de Melker HE, Spanjaard L, Schouls LM, van der Ende A (2006) Protection from routine vaccination at the age of 14 months with meningococcal serogroup C conjugate vaccine in the Netherlands. Pediatr Infect Dis J 25: 79–80. 16395110 10.1097/01.inf.0000195594.41449.c6

[30] Jennett B and Teasdale G (1981) Management of head injuries. Philadelphia: F.A.Davis.

[31] Saitou N, Nei M (1987) The neighbor-joining method: a new method for reconstructing phylogenetic trees. Mol Biol Evol 4: 406–425. 3447015 10.1093/oxfordjournals.molbev.a040454

[32] Tamura K, Dudley J, Nei M, Kumar S (2007) MEGA4: Molecular Evolutionary Genetics Analysis (MEGA) software version 4.0. Mol Biol Evol 24: 1596–1599. 17488738 10.1093/molbev/msm092

[33] Jolley KA, Maiden MC (2010) BIGSdb: Scalable analysis of bacterial genome variation at the population level. BMC Bioinformatics 11: 595. 21143983 10.1186/1471-2105-11-595 PMC3004885

