# Supplementary material for: Meningococcal Factor H Binding Protein fHbpd184 Polymorphism Influences Clinical Course of Meningococcal Meningitis
Source: PLoS One. 2012 Oct 23;7(10):e47973. doi: 10.1371/journal.pone.0047973 (PMC3479137; doi:10.1371/journal.pone.0047973)
Supplement: Table S2 — fHbp polymorphism of the 17 amino acids showing interaction with human factor H, according to fHbp subfamilies. (DOCX) [file pone.0047973.s002.docx]

**Table S2. fHbp polymorphism of the 17 amino acids showing interaction with human factor H, according to fHbp subfamilies**

|  | **fHBP subfamily** | |
| --- | --- | --- |
| **position^a^** | **B (212 isolates)** | **A (40 isolates)** |
| **Making salt bridge to fH** |  |  |
| 304 | E (acidic) | T (hydrophilic neutral; smaller side chain) |
| **Making hydrogen bonds to fH** |  |  |
| 180 | Q (hydrophilic neutral) | N (hydrophilic neutral; smaller side chain) |
| 181 | D (acidic) | N (hydrophilic neutral) |
| 183 | E (acidic) | D (acidic; smaller side chain) |
| 184 | H (basic; strongly polar) or D (acidic) | K (basic; strongly polar) |
| 185 | S (hydrophilic neutral) | I (hydrophobic; larger side chain) |
| 191 | K (basic; strongly polar) | Q (hydrophilic neutral; smaller side chain) |
| 193 | Q (hydrophilic neutral) or R (basic) | S (hydrophilic neutral; smaller side chain than Q and R) |
| 195 | R (basic) | L (hydrophobic neutral; smaller side chain) |
| 262 | Y (hydrophobic, weak acidic) or D (acidic) | E (acidic; smaller side chain than Y) |
| 274 | S (hydrophilic neutral) | L (hydrophobic neutral; larger side chain) |
| 286 | S (hydrophilic neutral) | T (hydrophilic neutral) |
| 306 | K (basic; strongly polar) or E (acidic) | K (basic; strongly polar) |

^a^Four of 17 aminoacids were conserved.
