# Supplementary material for: Meningococcal Factor H Binding Protein fHbpd184 Polymorphism Influences Clinical Course of Meningococcal Meningitis
Source: PLoS One. 2012 Oct 23;7(10):e47973. doi: 10.1371/journal.pone.0047973 (PMC3479137; doi:10.1371/journal.pone.0047973)
Supplement: Table S3 — The association constant (ka), dissociation constant (kd) and the thermodynamic dissociation constants (KD) of the binding between fH and fHbp, according to protein family. (DOCX) [file pone.0047973.s003.docx]

**Table S3. The association constant (k_a_), dissociation constant (k_d_) and the thermodynamic dissociation constants (K_D_) of the binding between fH and fHbp, according to protein family.**

|  | **Average binding values^a^ fHbp - fH** | |  |
| --- | --- | --- | --- |
|  | **fHbp family A** | **fHbp family B** | ***P*^b^** |
| k_a_ (M^-1^ s^-1^) | 0.80 x 10^5^ | 1.92 x 10^5^ | 0.04 |
| k_d_ (s^-1^) | 0.21 x10^-2^ | 2.87 x10^-2^ | 0.03 |
| K_D_ (nM) | 29 | 153 | 0.02 |

^a^Average values were calculated from Table 3 of Seib et al [22].

^b^Student T-test; two sided.
