# Supplementary material for: Meningococcal Factor H Binding Protein fHbpd184 Polymorphism Influences Clinical Course of Meningococcal Meningitis
Source: PLoS One. 2012 Oct 23;7(10):e47973. doi: 10.1371/journal.pone.0047973 (PMC3479137; doi:10.1371/journal.pone.0047973)
Supplement: Table S4 — Primers. (DOCX) [file pone.0047973.s004.docx]

**Table S4**. **Primers**

| **Primers used for *fHbp* sequencing** | |
| --- | --- |
| CH1870-1 | TGACCTGCCTCATTGATGC |
| CH1870-2 | CGGTAAATTATCGTGTTCGGACGGC |
| CH1870-3 | CAAATCGAAGTGGACGGGCAG |
| CH1870-4 | TGTTCGATTTTGCCGTTTCCCTG |
| CH1870-5 | GAAGTGGACGGACAAACCATC |
| **Primers used for amplification and mutagenesis of fHbp_10** | |
| 1P10DK F | gcaagtacaagactcggagAAAtccgggaagatggttgcg |
| 1P10DK R | cgcaaccatcttcccggaTTTctccgagtcttgtacttgc |
| NG2143 | CGCGGATCCCATATGGTTGCCGCCGACATCG |
| NG2145 | CCCGCTCGAGCTGCTTGGCGGCAAGAC |
